# P-1176. Optimal Agent and Timing of Surgical Antibiotic Prophylaxis and Surgical Site Infection in Pediatrics

**DOI:** 10.1093/ofid/ofae631.1362

**Published:** 2025-01-29

**Authors:** Martin T Tran, Shasta Erickson, Negar Ashouri, Melissa Powell, Laurie Moore, Tricia Morphew

**Affiliations:** CHOC Children's Hospital, Orange, California; CHOC Childrens, Orange, California; CHOC Children's Hospital, Orange, California; CHOC Childrens, Orange, California; CHOC Childrens, Orange, California; CHOC Childrens, Orange, California

## Abstract

**Background:**

Surgical site infections (SSI) make up ∼20% of all healthcare-associated infections (HAI), with 75% of SSI-associated deaths directly attributable to the SSI. Beta-lactams (BL) including cefazolin (CFZ) and cefoxitin (CFO) are recommended as surgical antibiotic prophylaxis (SAP). Cephalosporins are often avoided in patients with reported BL allergies despite cross-reactivity with penicillin at < 1%. Guidelines recommend giving SAP 60 min prior to incision. Cefazolin and cefoxitin pharmacokinetics (PK) exhibit rapid time to peak serum level and short elimination half-life. We aim to evaluate differences in SSI rate in patients who received CFZ or CFO vs. non-BL alternatives (BLA) and administration of preop BL dose at different time interval prior to incision.

**Figure:**
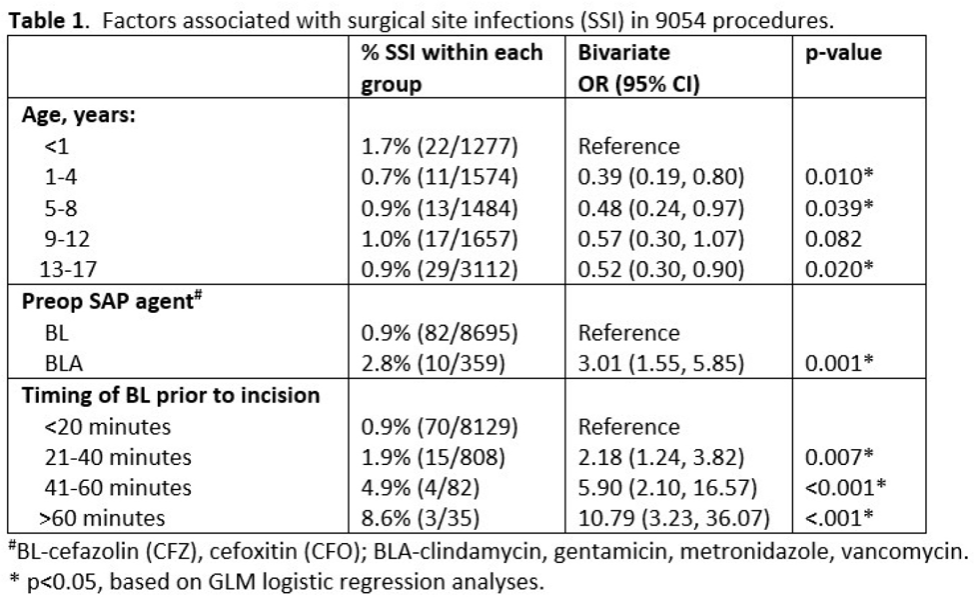

**Methods:**

Retrospective observational study included patients aged 0 – 17 years who received SAP at CHOC from Jul 2021 – Jun 2023. Outcomes include differences in SSI rate within 90 days of surgery between patients receiving recommended BL (CFZ, CFO) vs BLA (clindamycin, gentamicin, metronidazole, vancomycin). Rate of SSI in patients who received pre-op CFZ or CFO at different time interval (0-20 min, 21-40 min, 41-60 min) will be examined. Wound classification (I to IV) as defined by CDC. SSIs were defined according to National Healthcare Safety Network. Bivariate logistic regression analyses compare the odds of experiencing an SSI between study groups.

**Results:**

Median age was 9.0 years [IQR: 3.0, 14.0], 58.8% males. Wound classification is as follows: 37.6% (Class I), 34.1% (Class II), 22.9% (Class III), and 5.1% (Class IV). Overall SSI occurred in 1.0% (92/9054) of procedures. Beta-lactams were used as SAP in 96% of procedures, with 89.8% receiving pre-op dose within 20 min of incision. Laparoscopic appendectomy was the most common procedure at 10.9%. Age < 1 year, use of non-BL SAP and administration of SAP >20 min from incision was associated with significant increase in SSI (p< 0.05, Table 1). No difference in SSI rate observed in patients who continued receiving post-op SAP for class I-II procedures.

**Conclusion:**

Our study supports the continued use of CFZ, CFO compared to BLA for SAP. Pre-op doses of CFZ or CFO within 20 min of incision time were associated with decreased risk of SSI, likely due to their PK characteristics.

**Disclosures:**

**All Authors**: No reported disclosures

